# Characterization of transgene expression in adenoviral vector-based HIV-1 vaccine candidates

**DOI:** 10.1186/1743-422X-7-39

**Published:** 2010-02-18

**Authors:** Marie-Noëlle Takahashi, Judith A Rolling, Katherine E Owen

**Affiliations:** 1 Merck Manufacturing Division, Merck, West Point, PA 19486, USA

## Abstract

Recombinant adenovirus vectors have been extensively used in gene therapy clinical studies. More recently, the capability of inducing potent cell-mediated and humoral immunity has made these vectors equally attractive candidates for prophylactic or therapeutic vaccine applications. Merck and Co., Inc., developed HIV-1 vaccine candidates based on adenovirus serotype 5 (Ad5) vectors in which the E1 gene, a critical component for adenovirus replication, was replaced by the cytomegalovirus immediate/early promoter, followed by mutated versions of the HIV-1 gag, pol or nef genes (constructs referred to as MRKAd5gag, MRKAd5pol and MRKAd5nef, respectively). Vaccine performance was evaluated *in vitro *in a novel assay that measures the level of transgene expression in non-permissive A549 cells. Various combinations of vectors were studied. The results indicate that the vaccine induces a dose-dependent expression of the HIV-1 transgenes *in vitro*. Furthermore, the gag, pol, and nef transgenes are expressed differentially in A549 cells in an MOI-dependent and formulation-dependent manner, yielding an unexpected enhancement of protein expression in trivalent vs. monovalent formulations. Our data suggest that the presence of additional virus in multivalent formulations increases individual transgene expression in A549 cells, even when the amount of DNA encoding the gene of interest remains constant. This enhancement appears to be controlled at the transcriptional level and related to both the total amount of virus and the combination of transgenes present in the formulation.

## Findings

Recent clinical trials of Adenovirus-based HIV vaccines failed to demonstrate significant efficacy in protecting humans from HIV-1 infection or limiting viral load, despite strong pre-clinical immune response [1]. Nonetheless, in preparation for clinical studies, significant development took place to characterize these vaccines. In this case, we investigated the use of non-permissive A549 cells as an *in vitro *model for Adenovirus type 5-based gag, pol and nef transgene expression [2].

The ability of an Adenovirus-based vaccine to elicit a clinical response is dependent on its ability to deliver the appropriate transgene for expression in the vaccinee; therefore, determining the levels of transgene expression of a given vector can provide an appreciation of the efficiency with which the vector has delivered the transgene, offering a measure of the vaccine's relative *in vitro *potency [3-5]. A549 cells were chosen specifically for their inability to support recombinant Ad5 replication [6], such that all transgene expression would be the result of a single round of transgene delivery and transcription/translation. Expression of each of the three transgenes in this cell line was analyzed simultaneously by SDS-PAGE and ELISA, while Reverse Transcriptase PCR (RT-qPCR) was used to quantitate mRNA.

**Figure 1:**
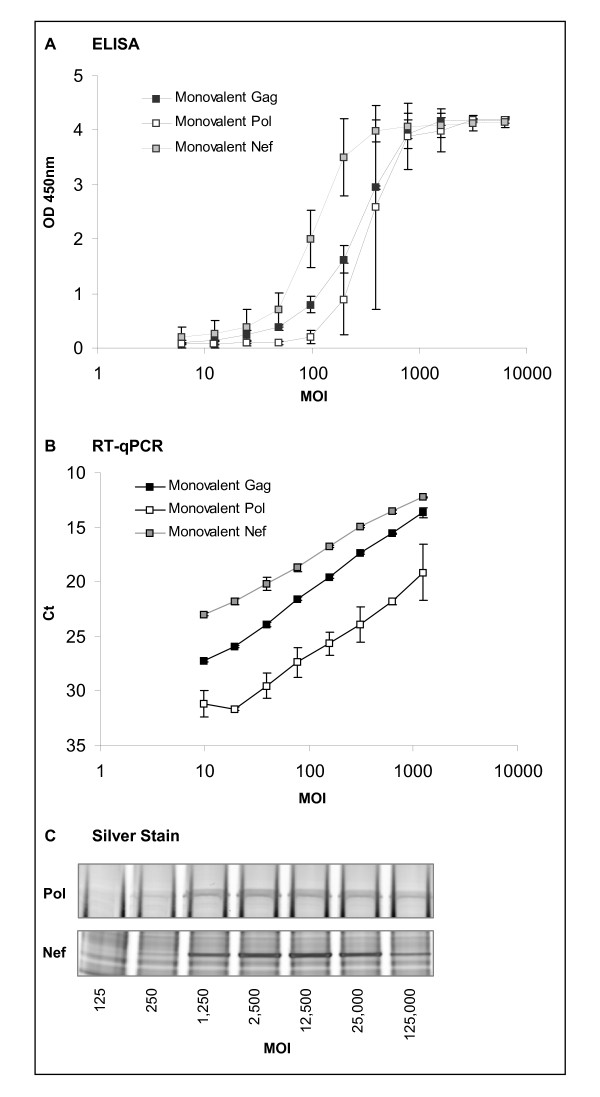
**Comparison of transgene expression levels in A549 cells infected with monovalent Ad5 vectors**. A549 cells were infected 48 hours post plant with various concentrations of monovalent Ad5 vectors in α-MEM media supplemented with 10% heat-inactivated FBS and 5% Pen/Strep. The cells were placed at 37°C/5% CO_2 _for 48 hours and then lysed with either a sodium deoxycholate/IGEPAL buffer for ELISA analysis or an RNA extraction buffer (RNeasy 96 Qiagen kit). The cell lysates were denatured and reduced prior SDS-PAGE analysis. Gag, pol and nef proteins were detected by colorimetric ELISA using HRP/TMB detection (A), gag, pol and nef mRNAs were detected by RT-qPCR (B). The protein profile of pol and nef proteins obtained by silver-stained SDS-PAGE is shown in (C). The MOI was determined by the quantity of infectious Ad5 particles measured by quantitative PCR based potency assay along with total Ad5 particles as measured by a genome quantitation assay. The error bars represent 95% confidence interval.

A549 cells were infected with monovalent formulations of MRKAd5gag, MRKAd5pol or MRKAd5nef vectors [2] at multiplicity of infections (MOI) ranging from 6 to 12,500 (Figure 1) [7,8]. As expected, ELISA (Figure 1A) and RT-qPCR (Figure 1B) both demonstrate an increase in antigen expression or transcription following increasing MOIs, within the dynamic range of each assay. Overall, a similar trend was observed throughout the different assays: the nef transgene appeared to be more dominantly expressed than the gag or pol transgenes in the MOI range of 100 to 1,000. Comparison of different gene products in a given assay platform must be made with caution since each gene product signal is dependent on the affinity of the detector (monoclonal antibodies for ELISA or primers/probes for RT-qPCR). A similar observation was made using a direct detection approach with SDS-PAGE followed by silver staining: analysis of infected cell lysates revealed that the nef protein was more intense than the pol protein (Figure 1C), suggesting that ELISA and RT-qPCR results were not an artifact of the detectors. Nef and pol band identities were confirmed by in-gel trypsin digestion and MALDI-TOF peptide mapping (data not shown). The 55-kDa gag protein could not be distinguished from host cell and FBS proteins.

**Figure 2:**
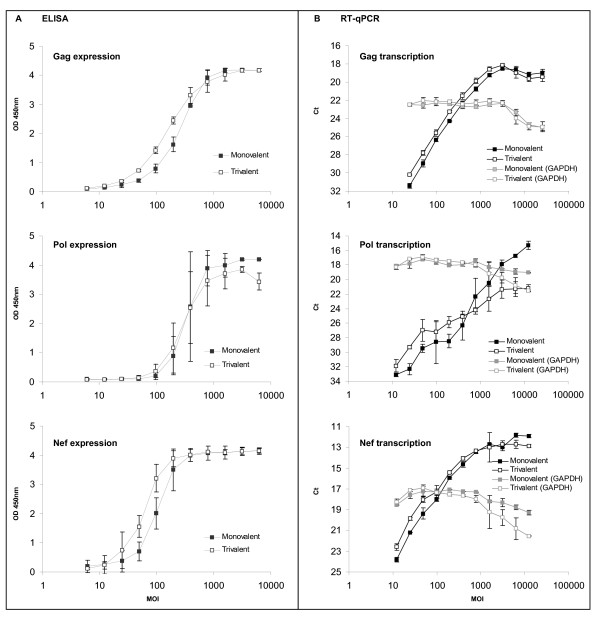
**Comparison of transgene expression in A549 cells infected with Ad5 monovalent or trivalent formulations**. A549 cells were infected as described in figure 1 with monovalent or trivalent Ad5 vectors. Gag, pol and nef proteins were detected by colorimetric ELISA (A), gag, pol and nef mRNAs were detected by RT-qPCR (B). The housekeeping gene GAPDH mRNA was monitored to address cell alteration (B). The MOI used at infection were normalized for transgene concentration, not for total virus concentration. The error bars represent 95% confidence interval.

In Phase I and Phase II clinical studies [2,9,10], the three constructs MRKAd5gag, MRKAd5pol and MRKAd5nef were administered simultaneously as a trivalent formulation. To compare each transgene's expression in the clinical *trivalent *formulation with the individual *monovalent *formulations, we assessed transgene transcription and expression levels in both formulations by ELISA and RT-qPCR (Figure 2). A549 cells were infected with either the monovalent or the trivalent formulations, normalizing the MOI for individual transgene concentration, not for total virus concentration, therefore allowing a direct comparison of the expression level of a given protein from a standard amount of genetic material. Surprisingly, the expression of gag and nef, in the linear range, appeared to be higher when the cells were infected with the trivalent formulation as compared with the monovalent formulation, as seen by a shift in the trivalent curves to the left (Figure 2A). These results were confirmed at the mRNA level (Figure 2B). Relative fold-increase for gag, pol and nef transcription and expression in trivalent relative to monovalent formulations were calculated by parallel line analysis (Table 1). On average, a 1.5-fold increase was observed for gag and nef at both the mRNA and protein levels, despite the fact that the total concentration of the monovalent gene in each formulation was constant. Pol expression by ELISA was roughly comparable whether the cells were infected with monovalent or trivalent formulations, but pol transcription was approximately 2-fold higher in cells infected with the trivalent formulation as compared with the monovalent formulation. The discrepancy observed for pol between the ELISA and RT-qPCR data might reflect a higher sensitivity of the RT-qPCR assay for this transgene. It is also possible that the ELISA developed for pol is less robust than the gag or nef ELISAs, presumably because the pol primary structure used by Merck was modified from the wild type form [1,11]. Consequently, the interaction of this modified pol with the commercially available antibodies may be suboptimal, rendering the ELISA less sensitive to improvements in expression levels.

**Table 1:** Fold-increase in transgene expression when comparing A549 cells infected with Ad5 trivalent relative to Ad5 monovalent formulations.

	Protein fold increase (95% CI)^a^	mRNA fold increase (95% CI)^b^
**gag**	1.5 (± 0.3)	1.5 (± 0.2)
**pol**	1.0 (± 0.1)	2.2 (± 0.2)
**nef**	1.7 (± 0.1)	1.6 (± 0.1)

^a ^Average (n = 2 independent experiments) and 95% Confidence Interval (95% CI) of gag, pol and nef protein fold increases determined by parallel line analysis on the full dose response curves.

^b ^Average (n = 3 independent experiments) and 95% Confidence Interval (95% CI) of RNA fold increases determined by parallel line analysis in the MOI ranges 25 -- 200 for gag and 12-100 for nef and pol.

To explain this transgene expression enhancement in the trivalent formulation we hypothesized that either the presence of additional transgenes impacted the transcription of each given gene or that the presence of additional viral load (3-fold higher in the trivalent formulation) somehow facilitated the overall transgene expression.

**Figure 3:**
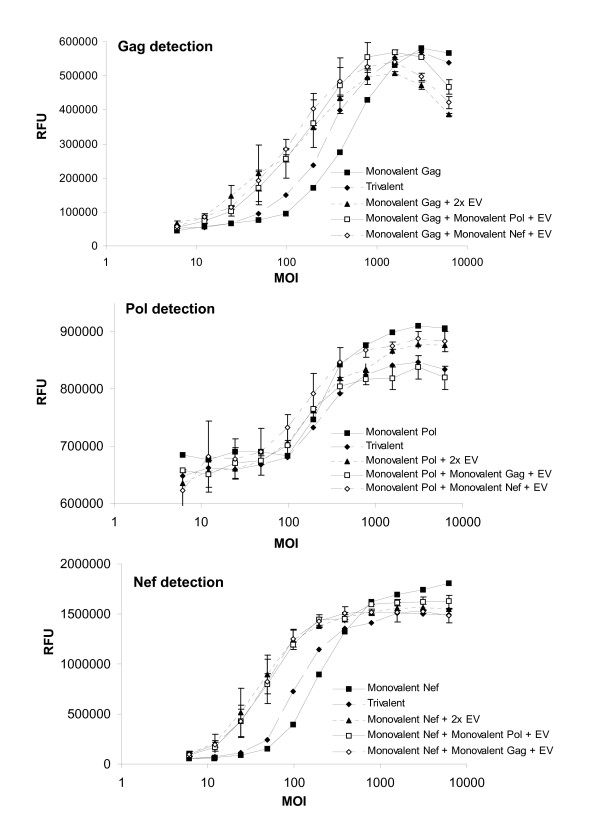
**Normalizing total virus concentration with Ad5 empty vectors and impact on transgene expression**. A549 cells were infected as described in figure 1. Detection of gag (A), pol (B), and nef (C) transgene expression in A549 cell lysates infected with monovalent, trivalent or multivalent formulations composed of monovalent vectors and empty vector to normalize total virus concentration. Fluorescence based ELISA signal is measured in relative fluorescence units (RFU). Similar results were observed on an independent set of experiments using OD_450 _detection (data not shown). The error bars represent 95% confidence interval (error bars for the monovalent and trivalent formulations were not available, n = 1).

Interestingly, at high MOI (>700 for ELISA and >1000 for RT-qPCR), more protein and mRNA were detected in cells infected with the monovalent formulations as compared with the trivalent formulations (Figure 2 and 3). Since trivalent formulations contain three times more virus than monovalent formulations, we evaluated the effect of this increase in viral concentration by measuring the steady-state transcription of a housekeeping gene, GAPDH, across the entire dose range of the RT-qPCR assay (Figure 2B). Cells infected with the trivalent formulation showed equivalent or less GAPDH transcription at high MOI than cells infected with the monovalent formulations. The levels of GAPDH mRNA began to decrease at MOIs ranging from approximately 700-1200, suggesting that signal decrease noted at the upper limits of the assay dose range is likely due to cell death caused by the virus. These data indicate that although transgene expression levels are higher in trivalent formulations, an *excessive *viral load likely compromises the cellular machinery, possibly leading to accelerated cell death.

To investigate whether the increase of transgene expression observed with the trivalent formulation was due to the higher amount of virus infecting the host cells or related to the simultaneous presence of one or more transgenes, we infected A549 cells with monovalent formulations of MRKAd5gag, MRKAd5pol or MRKAd5nef, supplemented either with "empty vectors" (EV, Ad5 constructs generated in the same way as the transgene-containing constructs, but missing the transgene cassette) or with a combination of EV and another monovalent vector to reach the equivalent number of virus particles that the cells were exposed to with the trivalent formulation. In this way, EV can normalize total non-replicating Ad5 virus concentration while leaving individual transgene concentration constant.

Surprisingly, ELISA analysis from cells infected with EV and either monovalent MRKAd5gag or MRKAd5nef revealed that the presence of EV in a given monovalent formulation dramatically boosted transgene expression, even exceeding the transgene expression level of the trivalent formulation (Figure 3). Whereas a 50% enhancement in transgene expression was observed in trivalent relative to monovalent formulations for gag and nef (determined by parallel line analysis on the full dose response curves), the addition of EV led to 190% and 270% increases in gag and nef transgene expression, respectively. Unlike gag and nef, no significant changes in pol transgene expression levels could be detected in the various infection combinations (Figure 3).

**Figure 4:**
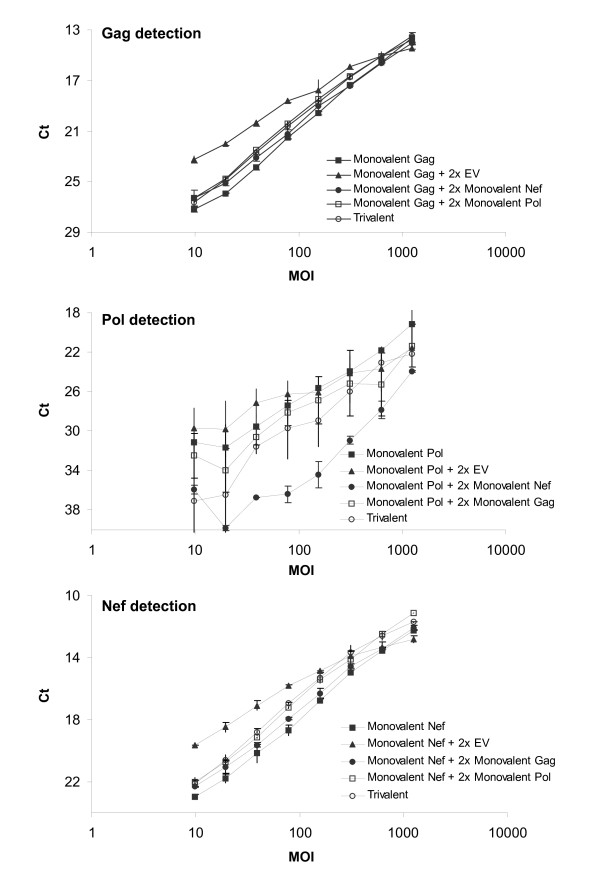
**Comparing the impact of Ad5 Empty Vectors vs. additional Ad5 transgenes on transgene transcription level**. RT-qPCR detection of gag (A), pol (B), and nef (C) transgene transcription in A549 cell lysates infected with monovalent, trivalent or multivalent formulations composed of monovalent vectors and empty vector to normalize total virus concentration. The error bars represent 95% confidence interval.

To confirm that the enhancement of transgene expression levels observed in the presence of EV was not transgene-dependent and that this enhancement occurs at the mRNA level, we compared transcription levels by RT-qPCR of A549 cells infected by monovalent Ad5 vectors supplemented with 2× EV or 2× of a specific Ad5 construct. The results confirmed that gag and nef transcriptions were maximal in conditions supplemented with EV, especially in the MOI range of 10 to 100, and minimal in the monovalent formulations (Figure 4). Overall enhancement of transcription with 2× EV was 320% for gag and almost 400% for nef, whereas the addition of 2× of a specific Ad5 construct did not differ significantly from the enhancement observed in the trivalent formulation (determined by parallel line analysis in the MOI ranges 10-78 of the dose response curves). These results confirm that the maximum enhancement previously observed derived from the presence of EV.

The data suggest that for an experimental adenovirus-based HIV-1 vaccine, transgene expression in non-permissive cells *in vitro *can be reproducibly modulated by the presence of additional replication-incompetent adenovirus. This modulation can occur in circumstances where another transgene-encoding adenovirus is present, as well as with the addition of adenovirus that does not contain the coding region for any transgene of interest. The mere presence of additional "empty" adenovirus particles devoid of transgene appears to enhance transgene expression. Our findings suggest these unexpected results may be worth consideration during dose and potency assay development for adenovirus-based therapeutics. Additional work is required to elucidate the mechanism of the observed transgene expression enhancement by the presence of additional adenoviral vectors. The data presented suggest that the mechanisms responsible for this enhancement are functioning prior to or during the transcription of the vector transgene. We hypothesize that the presence of additional virus results in an increase of cellular stress signals that activate transcription regulators such as MAP kinase and/or NFκB which in turn could up-regulate the CMV promoter activity driving transgene expression [12,13].

## Competing interests

All authors are employees of Merck, which paid for this study in its entirety.

## Authors' contributions

MNT carried out the RT-qPCR study, JAR carried out the ELISA study. KEO led the study. MNT, JAR and KEO wrote the manuscript. All authors read and approved the final manuscript.
